# Supplementary material for: Resolving the full spectrum of human genome variation using Linked-Reads
Source: Genome Res. 2019 Apr;29(4):635–45. doi: 10.1101/gr.234443.118 (PMC6442396; doi:10.1101/gr.234443.118)
Supplement: Supplemental Material [file supp_gr.234443.118_Supplemental_longranger-2.2.zip › longranger-2.2.tar.bz2!/lr2.2-public/lib/stan/cmdstan/stan/doc/api/html/math_2matrix_2variance_8hpp_source.html]

Stan: src/stan/math/matrix/variance.hpp Source File


|  |  |
| --- | --- |
|  | Stan  2.5.0  probability, sampling & optimization |


- Main Page
- Related Pages
- Namespaces
- Classes
- Files

- File List
- File Members

All Classes Namespaces Files Functions Variables Typedefs Enumerations Enumerator Friends Macros Pages

- src
- stan
- math
- matrix

variance.hpp

Go to the documentation of this file.

1 #ifndef STAN\_\_MATH\_\_MATRIX\_\_VARIANCE\_HPP

2 #define STAN\_\_MATH\_\_MATRIX\_\_VARIANCE\_HPP

3

4 #include <vector>

5 #include <boost/math/tools/promotion.hpp>

6 #include <stan/math/matrix/Eigen.hpp>

7 #include <stan/math/matrix/mean.hpp>

8 #include <stan/math/error\_handling/matrix/check\_nonzero\_size.hpp>

9

10 namespace stan {

11  namespace math {

12

21  template <typename T>

22  inline

23  typename boost::math::tools::promote\_args<T>::type

24  variance(const std::vector<T>& v) {

25  stan::math::check\_nonzero\_size("variance(%1%)",v,"v",(double\*)0);

26  if (v.size() == 1)

27  return 0.0;

28  T v\_mean(mean(v));

29  T sum\_sq\_diff(0);

30  for (size\_t i = 0; i < v.size(); ++i) {

31  T diff = v[i] - v\_mean;

32  sum\_sq\_diff += diff \* diff;

33  }

34  return sum\_sq\_diff / (v.size() - 1);

35  }

36

43  template <typename T, int R, int C>

44  inline

45  typename boost::math::tools::promote\_args<T>::type

46  variance(const Eigen::Matrix<T,R,C>& m) {

47  stan::math::check\_nonzero\_size("variance(%1%)",m,"m",(double\*)0);

48

49  if (m.size() == 1)

50  return 0.0;

51  typename boost::math::tools::promote\_args<T>::type

52  mn(mean(m));

53  typename boost::math::tools::promote\_args<T>::type

54  sum\_sq\_diff(0);

55  for (int i = 0; i < m.size(); ++i) {

56  typename boost::math::tools::promote\_args<T>::type

57  diff = m(i) - mn;

58  sum\_sq\_diff += diff \* diff;

59  }

60  return sum\_sq\_diff / (m.size() - 1);

61  }

62

63  }

64 }

65 #endif

stan::math::variance

boost::math::tools::promote\_args< T >::type variance(const std::vector< T > &v)

Returns the sample variance (divide by length - 1) of the coefficients in the specified standard vect...

**Definition:** variance.hpp:24

mean.hpp

stan::math::mean

boost::math::tools::promote\_args< T >::type mean(const std::vector< T > &v)

Returns the sample mean (i.e., average) of the coefficients in the specified standard vector...

**Definition:** mean.hpp:23

Eigen.hpp

check\_nonzero\_size.hpp

stan::math::check\_nonzero\_size

bool check\_nonzero\_size(const char \*function, const T\_y &y, const char \*name, T\_result \*result)

Return true if the specified matrix/vector is of non-zero size.

**Definition:** check\_nonzero\_size.hpp:33


---

[ Stan Home Page ]

*© 2011–2014,
Stan Development Team.*
